# Clinical Management of Argentine Hemorrhagic Fever using Ribavirin and Favipiravir, Belgium, 2020

**DOI:** 10.3201/eid2607.200275

**Published:** 2020-07

**Authors:** Ioannis Veliziotis, Alain Roman, Delphine Martiny, Gerlind Schuldt, Marc Claus, Nicolas Dauby, Sigi Van den Wijngaert, Charlotte Martin, Rakan Nasreddine, Claudia Perandones, Romain Mahieu, Corien Swaan, Serge Van Praet, Deborah Konopnicki, Maria A. Morales, Denis Malvy, Etienne Stevens, Philippe Dechamps, Erika Vlieghe, Olivier Vandenberg, Stephan Günther, Michèle Gérard

**Affiliations:** Université de Bruxelles, Brussels, Belgium (I. Veliziotis, A. Roman, D. Martiny, M. Claus, N. Dauby, S. Van den Wijngaert, C. Martin, R. Nasreddine, D. Konopnicki, E. Stevens, P. Dechamps, O. Vandenberg, M. Gérard);; Saint-Pierre University Hospital, Brussels (I. Veliziotis, A, Roman, M. Claus, N. Dauby, C. Martin, R. Nasreddine, S. Van Praet, D. Konopnicki, E. Stevens, P. Deschamps, M. Gérard);; Bernhard Nocht Institute for Tropical Medicine, Hamburg, Germany. (G. Schuldt, S. Günther);; National Administration of Laboratories and Institutes of Health, Buenos Aires, Argentina (C. Perandones);; Common Community Commission, Brussels (R. Mahieu);; National Institute for Public Health and the Environment, Bilthoven, the Netherlands (C. Swaan);; Instituto Nacional de Enfermedades Virales Humanas, Pergamino, Argentina (M.A. Morales);; World Health Organization Collaborating Centre for Reference and Research of Arbovirus and Hemorrhagic Fever Virosis, Pergamino (M.A. Morales);; University Hospital Center of Bordeaux, Bordeaux, France (D. Malvy);; University Hospital Antwerp, Belgium (E. Vlieghe);; University College London, London, UK (O. Vandenberg)

**Keywords:** Junin virus, JUNV, New World arenaviruses, viruses, Argentine hemorrhagic fever, travel, ribavirin, favipiravir, public health, clinical management, containment, antiviral drugs, Belgium

## Abstract

We report a case of Argentine hemorrhagic fever diagnosed in a woman in Belgium who traveled from a disease-endemic area. Patient management included supportive care and combination therapy with ribavirin and favipiravir. Of 137 potential contacts, including friends, relatives, and healthcare and laboratory workers, none showed development of clinical symptoms of this disease.

Argentine hemorrhagic fever (AHF) is a severe hemorrhagic fever caused by a New World arenavirus, Junin virus (JUNV), which was discovered in 1958 (*1*). The virus reservoir consists of rodents found in humid pampas in South America. The endemic area covers 150,000 km^2^ distributed over 4 provinces in Argentina; ≈5.6 million persons are at risk (*2*).

Until 1992, the year when a prophylactic vaccine was introduced, annual outbreaks affected mainly male agricultural workers (*2*). The number of confirmed cases reported annually has decreased from 400–500 before 1992 to 13 cases in 2018 (*3*).

In January 2020, AHF was diagnosed in a woman in Brussels, Belgium, who had traveled from Argentina to Europe. We report clinical manifestations, management, and public health response for this case.

## The Study

In early January 2020, a 41-year old woman was admitted to the hospital in Brussels because of lethargy, confusion, and fever. She had been in Argentina and arrived in Amsterdam, the Netherlands, by a connecting flight from Madrid. After 4 days in Amsterdam, she traveled by bus to Brussels.

At admission, her travel partner reported that she had an influenza-like illness (including headache, myalgia, anorexia, and sore throat) since 1 week before admission. The patient had consulted a physician before departure from Argentina. The physician performed a blood sample analysis that showed the following results: hemoglobin 16 g/dL, leukocyte count 3,200 cells/mm^3^, and platelet count 144,000 cells/mm^3^. Episodes of vomiting at home were reported during her stay in Amsterdam.

In the emergency department, the patient had a seizure. Physical examination showed no petechia or purpura, but vaginal bleeding was observed. Emergency department laboratory results indicated thrombocytopenia, leukopenia, and severe rhabdomyolysis (Table 1). Results of whole-body computed tomography scan were unremarkable. A lumbar puncture was performed, and results of cerebrospinal fluid analysis were within reference ranges. The patient was admitted to the intensive care unit (ICU), and empirical antimicrobial drug therapy with ceftriaxone was started.

**Table 1:** Laboratory findings for a patient with Argentine hemorrhagic fever, Belgium, 2020*

Parameter	Reference ranges/values	Day 1 (ED)	Day 2 (ICU)	Day 9	Day 21	Day 43
Hemoglobin, g/dL	12.0–16.0	15.1	12.3	6.1	7.2	9.3
Erythrocytes, x 10^6^/μL	3.80–5.00	5.2	4.3	2.12	2.6	3.2
Hematocrit, %	35.0–47.0	43.1	35.2	NA	22.8	30.8
Platelets, x 10^3^/μL	150–440	66	48	76	268	350
Leukocytes, x 10^3^/μL	3.50–11.00	1.48	0,81	4	0.9	5.8
Neutrophils, %	40.0–75.0	72.5	61,7	80.5	NA	40.4
Lymphocytes, %	2.0–10.0	12.4	NA	11	NA	41.9
CRP, mg/L	<5.0	3.1	2,4	60.9	137	5
Schistocytes/1,000 RBC	<10	3	4	NA	NA	NA
Haptoglobin, mg/dL	30–200	NA	<10	NA	NA	NA
Serum iron, μg/dL	50–170	NA	163	NA	NA	NA
Serum ferritin, μg/L	30–200	NA	36,209	NA	NA	NA
aPTT, s	18.7–32.1	38.8	38,3	NA	NA	NA
INR, s	0.95–1.31	1.19	1.34	NA	NA	NA
Fibrinogen, mg/dL	150–400	138	100	NA	NA	NA
d-dimer, ng/mL	0–500	NA	>4,500	NA	NA	NA
AST, U/L	<32	1416	1,502	518	72	NA
ALT, U/L	<33	238	245	94	48	NA
ALP, U/L	35–104	165	187	NA	NA	NA
γ-GT, U/L	6–42	115	193	NA	NA	NA
LDH, U/L	135–214	NA	2,359	NA	NA	NA
Total bilirubin, mg/dL	<1.2	NA	0,6	3.9	2.4	1.1
Creatinine kinase, U/L	26–192	NA	7,341	2,363	15	28
Triglycerides, mg/dL	<150	NA	135	NA	NA	NA
C3, g/L	0.80–1.64	NA	0.3	NA	NA	NA
C4, g/L	0.10–0.40	NA	0.24	NA	NA	NA
HIV	NA	NA	Negative	NA	NA	NA
Yellow fever IgG/IgM	NA	NA	Negative	NA	NA	NA
Dengue virus IgG/IgM	NA	NA	Negative	NA	NA	NA
*Leptospira* sp.	NA	NA	Negative	NA	NA	NA
Chikungunya IgG/IgM	NA	NA	Negative	NA	NA	NA
Hantavirus IgG/IgM	NA	NA	Negative	NA	NA	NA
Malaria	NA	Negative	Negative	NA	NA	NA

*ALP, alkaline phosphatase; ALT, alanine aminotransferase; aPTT, activated partial thromboplastin time; AST, aspartate aminotransferase; CRP, C-reactive protein; ED, emergency department; γ-GT, γ-glutamyltransferase; ICU, intensive care unit; INR, international normalized ratio; LDH, lactate dehydrogenase; NA, not available; RBC, red blood cells.

We obtained blood culture, and results became positive the next day for *Escherichia coli*. Bone marrow aspiration examination was compatible with hemophagocytic lymphohisticytosis. The early stage of the patient’s hospital course was marked by fluctuating fever, ginigival bleeding, and bleeding at the site of the central venous catheter. We managed the seizure by using levetiracetam. At this stage, a diagnosis of AHF was being considered as a possible explanation for the patient’s illness.

On day 3 of hospitalization, we identified the city of origin for the patient as Perez, Argentina, which is located in Santa Fe Province. After consultation with experts from Argentina (C.P. and M.A.M.), AHF was considered as highly possible. Consequently, a confirmatory blood sample was sent to the World Health Organization Collaborating Center for Arbovirus and Hemorrhagic Fever Reference and Research at the Bernhard Nocht Institute for Tropical Medicine in Hamburg, Germany. Treatment with oral ribavirin was initiated because the intravenous form of this drug was not available.

On day 5 of hospitalization, the patient was confirmed to be positive for JUNV by real-time PCR and conventional reverse transcription PCR (RT-PCR) (*4*). The cycle threshold in the real-time RT-PCR was 18, indicating a high viral load. JUNV infection was confirmed by Sanger sequencing of the amplicons. Intravenous ribavirin was not available immediately but was available the next day. This drug was given orally and then intravenously the next day. The patient had to be intubated because of airway obstruction secondary to tongue hematoma and epistaxis.

The next day, because of its antiviral activity in JUNV infection animal models (*5*,*6*) and previous experience with Lassa fever (*7*), oral favipiravir was given through a nasogastric tube (2,000 mg loading dose, followed by 1,200 mg 2×/d) (Figure, panel A). Hemolytic anemia secondary to ribavirin therapy resulted in its discontinuation on day 9. Because of persistently high viral load, the dose of favipiravir was progressively increased (to 1,800 mg 2×/d) and finally stopped after 14 days.

**Figure:**
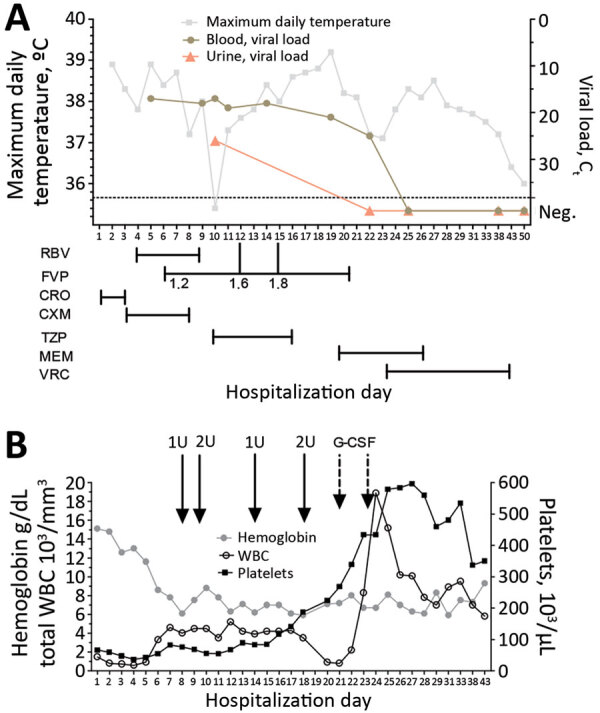
Biologic and virologic evolution in relation to treatment and supportive care for a patient with Argentine hemorrhagic fever, Belgium, 2020. A) Evolution of maximum daily temperature and blood and urine viral load during hospitalization. Antiviral and antimicrobial drug treatment scheme are shown. Dosages of FVP are indicated in grams. B) Evolution of hemoglobin, total leukocytes, and platelets during hospitalization. Solid arrows indicate administration of red blood cell units, and dashed arrows indicate administration of G-CSF. C_t_ values <40 indicate undetectable viral load. CRO, ceftriaxone; C_t_, cycle threshold; CXM, cefuroxime; FVP, favipiravir; G-CSF, granulocyte colony-stimulating factor; MEM, meropenem; Neg., negative; RBV, ribavirin; TZP, piperacillin/tazobactam; VRC, voriconazole; WBC, white blood cells.

Throughout her time in the ICU, the patient received broad-spectrum antimicrobial drugs for ventilator-associated pneumonia, voriconazole for probable invasive pulmonary aspergillosis, and filgrastim for persistent neutropenia (Figure, panel B). The patient was successfully extubated on day 23 of hospitalization and discharged from the hospital after 50 days without sequelae.

All residual samples for the patient stored in the laboratory were traced and destroyed. Starting from when the patient first came to our hospital, all healthcare workers who came in contact with the patient used gloves, gowns, and filtering facepiece 2 respiratory masks. Needlestick or sharp injuries were not reported. Starting from day 7 because of signs of bleeding, enhanced personal protective equipment was used in the ICU, including full-body suits and powered air-purifying respirators. Donning and doffing procedures were followed and included observation by another staff member. Body fluids from the patient were gelified and inactivated with peracetic acid. Standard biosecurity measures were applied for laboratory workers until day 4. Afterward, all laboratory investigations were conducted in a Biosafety Level 3 laboratory with enhanced personal protective equipment.

We conducted contact tracing for all healthcare workers who had come in contact with the patient or her samples before the diagnosis of AHF. Public health authorities from the 3 countries in Europe involved conducted contract tracing activities among all potential contacts, including family and friends. In Amsterdam, 2 low-risk contacts were identified among those who had cleaned the apartment in which the patient stayed. According to the European Centre for Disease Prevention and Control Risk Assessment Guidelines for Infectious Diseases Transmitted on Aircraft for viral hemorrhagic fever (*7*), fellow airline and bus passengers were not considered to be at risk because the patient had not begun to show symptoms of bleeding, vomiting, diarrhea, or urine loss at that time.

We classified contacts as high risk (n = 77) or low risk (n = 60) according to the extent of exposure to body fluids of the patient (Table 2). All contacts were notified by telephone or mail and asked to monitor themselves for fever for 21 days from the day of most recent exposure (upper limit of incubation period). During the surveillance period, 1 high-risk contact became symptomatic (fever) but was ultimately given a diagnosis of infection with influenza A virus. Testing for AHF was not performed. We identified no clinically apparent secondary cases of AHF among the 137 contacts.

**Table 2:** Contact with Junin virus for laboratory personnel, healthcare workers, and relatives for a patient with Argentine hemorrhagic fever, Belgium, 2020*

Contact, generic description	High risk	Low risk	No risk
Laboratory			
Present in laboratory where patient's blood was processed			X
Touching, moving closed blood tube			X
Opening blood tube without touching sample		X	
Pipetting or other sample handling in biosafety cabinet with gloves		X	
Pipetting or other sample handling without gloves or not in biosafety cabinet	X		
Microscopy of wet sample or preparing thick smear outside biosafety cabinet	X		
Microscopy of dried or fixated sample outside biosafety cabinet		X	
Preparing smears, including thick smear in biosafety cabinet, with gloves		X	
Serologic test outside biosafety cabinet	X		
Discarding samples in waste bucket on floor	X		
Rinsing cell counting chambers and other reused materials	X		
Care giver			
Brief presence in patient room, without touching anything			X
Examining the patient and using gloves, mask, or glasses		X	
Examining the patient and not using gloves, mask, or glasses	X		
Drawing blood or handling other body fluids with gloves, mask, glasses		X	
Drawing blood or handling other body fluids without gloves, mask, or glasses	X		
Resuscitating patient and using gloves, mask, glasses		X	
Invasive procedure (catheter placement, puncture, lumbar puncture) with gloves, mask, glasses		X	
General			
Sexual contact	X		
Household contact with patient's body fluids	X (Brussels, Belgium)	X (Amsterdam, the Netherlands)	
Having been near patient without contact with body fluids			X

*High risk indicates unprotected contact (skin, mucosa) with body fluids or aerosols, not wearing intact PPE; low risk indicates protected contact with patient or body fluids, wearing intact and correct PPE; no risk indicates no contact with patient of body fluid. X indicates to which group the contact in the first column belongs. PPE, personal protective equipment.

## Conclusions

This case illustrates the possibility of imported New World arenavirus hemorrhagic fever outside South America. Interruption of vaccine production has been reported in Argentina and if persistent, this interruption could increase the number of persons at risk for contracting the disease (*3*). This patient was not vaccinated and did not engage in any agricultural activities. The only identifiable at-risk activity was jogging on a dirt road next to her house.

Immune plasma obtained from convalescent-phase patients and containing neutralizing antibody has been shown to decrease the mortality rate from 30% to 1% if initiated within the first 8 days of disease (*8*). The delay between initial symptomatology and AHF diagnosis (13 days) was considered too long to administer immune plasma to the patient. The National Institute of Human Viral Diseases of Argentina provided us with immune plasma matched to blood groups of 3 high-risk contacts identified among the relatives of the patient. Because none of them showed development of illness, immune plasma was ultimately not administered.

Nosocomial transmission of New World arenavirus causing hemorrhagic fever has been reported (*9*). For this patient, we identified no secondary case among healthcare workers and laboratory personnel, confirming the low infectious risk for infection with JUNV when appropriate biosafety and personal protection measures are taken.
